# Non-invasive fetal electrocardiography, electrohysterography and speckle-tracking echocardiography in the second trimester: study protocol of a longitudinal prospective cohort study (BEATS-study)

**DOI:** 10.1186/s12884-021-04265-8

**Published:** 2021-11-25

**Authors:** T. J. Nichting, M. W. E. Frenken, D. A. A. van der Woude, N. H. M. van Oostrum, C. M. de Vet, B. G. van Willigen, J. O. E. H. van Laar, M. van der Ven, S. G. Oei

**Affiliations:** 1 Department of Gynaecology and Obstetrics, Máxima MC, P.O. Box 7777, 5500 MB Veldhoven, The Netherlands; 2 Department of Electrical Engineering, Eindhoven University of Technology, P.O. Box 513, 5600 MB Eindhoven, The Netherlands; 3 Eindhoven MedTech Innovation Centre, P.O. Box 513, 5600 MB Eindhoven, The Netherlands; 4 Department of Gynaecology and Obstetrics, University Hospital Gent, 9000 Gent, Belgium

**Keywords:** Non-invasive fetal electrocardiography, Electrohysterography, Speckle-tracking echocardiography, Hypertensive disorders of pregnancy, Fetal growth restriction, Preterm birth, Heart rate variability, Cardiac time intervals, Cardiotocogram, Antepartum monitoring

## Abstract

**Background:**

Worldwide, hypertensive disorders of pregnancy (HDP), fetal growth restriction (FGR) and preterm birth remain the leading causes of maternal and fetal pregnancy-related mortality and (long-term) morbidity. Fetal cardiac deformation changes can be the first sign of placental dysfunction, which is associated with HDP, FGR and preterm birth. In addition, preterm birth is likely associated with changes in electrical activity across the uterine muscle. Therefore, fetal cardiac function and uterine activity can be used for the early detection of these complications in pregnancy. Fetal cardiac function and uterine activity can be assessed by two-dimensional speckle-tracking echocardiography (2D-STE), non-invasive fetal electrocardiography (NI-fECG), and electrohysterography (EHG). This study aims to generate reference values for 2D-STE, NI-fECG and EHG parameters during the second trimester of pregnancy and to investigate the diagnostic potential of these parameters in the early detection of HDP, FGR and preterm birth.

**Methods:**

In this longitudinal prospective cohort study, eligible women will be recruited from a tertiary care hospital and a primary midwifery practice. In total, 594 initially healthy pregnant women with an uncomplicated singleton pregnancy will be included. Recordings of NI-fECG and EHG will be made weekly from 22 until 28 weeks of gestation and 2D-STE measurements will be performed 4-weekly at 16, 20, 24 and 28 weeks gestational age. Retrospectively, pregnancies complicated with pregnancy-related diseases will be excluded from the cohort. Reference values for 2D-STE, NI-fECG and EHG parameters will be assessed in uncomplicated pregnancies. After, 2D-STE, NI-fCG and EHG parameters measured during gestation in complicated pregnancies will be compared with these reference values.

**Discussion:**

This will be the a large prospective study investigating new technologies that could potentially have a high impact on antepartum fetal monitoring.

**Trial registration:**

Registered on 26 March 2020 in the Dutch Trial Register (NL8769) via https://www.trialregister.nl/trials and registered on 21 October 2020 to the Central Committee on Research Involving Human Subjects (NL73607.015.20) via https://www.toetsingonline.nl/to/ccmo_search.nsf/Searchform?OpenForm.

## Background

Hypertensive disorders of pregnancy (HDP), fetal growth restriction (FGR) and preterm birth are three major complications of pregnancy, associated with high maternal and fetal morbidity and mortality. These pregnancy complications have incidences of respectively up to 15 [1], 10 and 18% [2]. They often occur in combination because of an overlapping pathophysiology between HDP, FGR and preterm birth; for this study protocol merged as pregnancy-related diseases (PRD).

The contemporary method for antepartum fetal and uterine monitoring is cardiotocography (CTG), using Doppler ultrasound (DU) and external tocodynamometry (TOCO), in combination with abdominal ultrasound measurements evaluating fetal growth and uteroplacental blood flow. However, none of these tools has been truly successful for the early detection of PRD. Therefore, there is a need for an antepartum monitoring method that can detect PRD at an early stage of pregnancy, even before clinical symptoms are perceptible, to comprehensively monitor the pregnancy and prevent severe complications.

The pathophysiology of PRD is complex, but abnormal placentation has an important place. Normal placentation is associated with cytotrophoblast invasion in the spiral arteries causing structural changes which a decrease of the resistance of these vessels. This process makes the spiral arteries less sensitive to vasoconstriction, which ensures an undisturbed exchange function between mother and child. In HDP, normal cytotrophoblast invasion has been disrupted which induces higher sensitivity to vasoconstriction, which may lead to chronic placental ischemia [3]. FGR frequently evolves as a consequence of chronic placental ischemia, consequently failing to supply fetal needs for growth and development [3].

The chronic hypoxic environment and elevated placental resistance leads to fetal cardiac remodelling, initiated by pressure changes and volume overload of the fetal heart. To maintain cardiac output, the ventricle shape of the fetal heart changes from an ellipse to a more globular shape and myocardial hypertrophy occurs [4–6].

Abnormal placentation also induces a decrease in the placental enzyme 11 Beta Hydroxysteroid Dehydrogenase (11β-HSD) type 2 that normally acts as a dehydrogenase, converting cortisol into the inactive metabolite cortisone [7]. This process is physiologic at term to inhibit fetal growth and induce labour [8]. However, during the second trimester a decrease of 11β-HSD type 2 can be a sign of abnormal placentation as present in HDP or FGR [9, 10]. Low levels of the placental enzyme 11β-HSD type 2 result in an increased exposure of the fetus to cortisol [7]. This hypothetically leads to overstimulation of the sympathetic branches of the autonomic nervous system. Subsequently, a sympathetic-parasympathetic imbalance is caused that can be reflected by a decreased fetal heart rate variability (fHRV).

Spontaneous preterm delivery is likely associated with a change in uterine cell excitability, favouring conduction of electrical activity across the uterine muscles [11, 12]. High cortisol levels prepare the fetus for extra-uterine life by fetal lung maturation [13]. Cortisol also stimulates the expression of corticotropin releasing hormone (CRH) in the placenta, the major source of CRH secretion from 16 weeks GA onwards [13, 14]. In the fetus, increased CRH leads to a positive feed-forward loop that further drives the hypothalamic-pituitary-adrenal axis. Ultimately, this results in the start of labour by contraction-associated protein activation and stimulation of uterotonic agonists [13]. Moreover, CRH directly affects myometrial contractility [13, 15]. In this way, the altered uterine cell excitability in preterm birth may be a result of high fetal cortisol levels in PRD.

With the above in mind, promising novel technologies for evaluation of fetal cardiac functioning and uterine activity are two-dimensional speckle-tracking echocardiography (2D-STE), non-invasive fetal electrocardiography (NI-fECG) and electrohysterography (EHG). 2D-STE is an echocardiographic imaging technique that analyses tissue motion in the heart by using the natural occurring speckle pattern in the myocardium, 2D-STE is therefore able to assess myocardial deformation [16, 17]. Deformation measurements are thought to change with fetal cardiac adaptation caused by placental dysfunction [18–20]. By using the Nemo Fetal Monitoring System® (NFMS), a non-invasive recording method using a transabdominal electrode patch, NI-fECG and EHG can be measured. NI-fECG reflects fHRV by measuring a beat-to-beat heart rate [21], whereas EHG measures uterine electrical activity. Combining NI-fECG and EHG can also display a contemporary CTG.

All 2D-STE, NI-fECG and EHG might be helpful to detect PRD at a very early stage of pregnancy. However, more extensive research is required to enhance their diagnostic potential and clinical applicability. Therefore, this study aims to gain new expertise on normal fetal cardiac functioning and uterine activity in the second trimester, and to investigate the diagnostic potential of 2D-STE, NI-fECG and EHG for early detection of PRD.

## Methods

This paper is based on version 4.0 of the study protocol approved by the medical ethics committee.

### Study objectives

In order to detect PRD based on 2D-STE or NI-fECG and EHG parameters, it is first needed to better understand the physiological changes of the fetal heart and uterine muscle in early pregnancy. Therefore, the primary objective is to generate reference values of 2D-STE, NI-fECG and EHG parameters in uncomplicated pregnancies during the second trimester (16 until 28 weeks gestational age (GA)), providing more detailed information about the (electro)physiological development and modelling of the fetal heart and uterine activity.

Secondary, PRD manifestation will be related to 2D-STE, NI-fECG and EHG parameters to investigate the diagnostic potential of these parameters and the possibility to develop a prediction model.

Additional study objectives will be: to explore cardiac fetal-maternal coupling as maternal ECG can be derived from the NFMS simultaneously with NI-fECG. Moreover, the collected data can be used to tune and verify a mathematical model that simulates fetal development in a perinatal life support system that is currently being developed. Finally, a quality assessment will be performed evaluating the inter- and intra-observer variability, reliability and reproducibility of 2D-STE and NI-fECG measurements.

### Setting

For this longitudinal prospective cohort study, pregnant women will be recruited from a tertiary care hospital and a primary midwifery practice, both situated in Veldhoven, The Netherlands. The women will be recruited around 12 weeks GA during a regular consultation with their obstetric healthcare professional, e.g. gynaecologist, resident or midwife.

### Population

To be eligible to participate in this study, woman must be 18 years or older and pregnant with an uncomplicated singleton pregnancy before 16 weeks GA at the time of inclusion (Table 1).

**Table 1:** In- and exclusion criteria

Inclusion criteria	Exclusion criteria
Pregnant with a singleton pregnancy.	Pre-existing maternal disease that might influence fetal development (e.g. diabetes mellitus, pre-existent hypertensive disease or chronic medication use for maternal illness).
Uncomplicated pregnancy at the time of inclusion.	Abnormalities found at any ultrasound scan that might influence fetal cardiac function (e.g. fetal cardiac arrhythmias or congenital cardiac abnormalities).
GA <16+0 weeks	Contra-indications to abdominal patch placement of the NFMS (e.g. dermatologic diseases of the abdomen precluding preparation of the abdomen with abrasive paper, or external or implanted electrical stimulator).
Oral and written consent is obtained.	Insufficient ability in understanding Dutch or English language.
Maternal age ≥ 18 years.	

*GA* gestational age, *NFMS* Nemo Fetal Monitoring System®

Exclusion criteria are a pre-existing maternal disease that might influence fetal development; fetal abnormalities that might influence fetal cardiac functioning found at any ultrasound scan during pregnancy; contra-indications to abdominal patch placement or insufficient ability to understand Dutch or English language (Table 1).

Included participants can withdraw from study participation at any time without giving reason and without any consequences. The researcher can also decide to withdraw a woman from the study for urgent medical reasons making measurements impossible and unsafe (e.g. necessity for hospitalisation).

Regular pregnancy follow-up continues at the women’s obstetric healthcare professional. The researcher will evaluate if a woman developed a PRD during pregnancy, after childbirth. This information will be obtained from the patient record at the tertiary care hospital or primary midwifery practice. If the woman did develop a PRD, all measurements will be evaluated in the corresponding PRD group. If the woman did not develop a PRD, the data will be evaluated in the control group. To increase internal validity, unambiguous definitions for PRD are formulated (Table 2).

**Table 2:** Definitions of PRD

PRD	Definition
HDP [1]	
*Pregnancy induced hypertension*	Two independent measurements (time gap at least 4 hours) after 20 weeks GA of a systolic blood pressure ≥140mmHg **AND/OR** a diastolic blood pressure ≥90mmHg (severe hypertension = systolic blood pressure ≥160mmHg, **AND/OR** diastolic blood pressure ≥110mmHg).
*Pre-eclampsia*	- Hypertension as described above **AND** one or more of the following: - Proteinuria **AND/OR**: ○ Protein/creatinine ratio ≥30mg/mmol **OR**; ○ Proteinuria of ≥300mg/24 hour. (Massive proteinuria = protein/creatinine ratio >500mg/mmol **OR** >5g/24 hour) - Uteroplacental dysfunction (such as FGR, abnormal umbilical artery Doppler, or stillbirth) **AND/OR**: - Other maternal organ dysfunction, including: ○ Acute kidney injury (creatinine ≥ 90μmol/L). ○ Liver involvement (elevated transaminases e.g. ALAT or ASAT > 40U/L). ○ Neurological complications (eclampsia, altered mental state, blindness, stroke, clonus, severe headaches, persistent visual scotoma). ○ Haematological complications (thrombocytopenia <150,000/μL, DIC, haemolysis).
*HELLP-syndrome*	Combination of symptoms that signifies a more serious manifestation of pre-eclampsia [1]. Defined as a combination of: - Haemolysis (LDH ≥600 U/L, haptoglobin <0.2g/L **AND**; - Elevated liver enzymes (ASAT or ALAT >70U/L) **AND**; - Low platelets (<100*10^9^/L).
FGR [22]	Birthweight corrected for GA <10th percentile.
Preterm birth [23]	Babies born alive <37 weeks GA. - Extremely preterm: <28 weeks GA. - Very preterm: 28 to 32 weeks GA. - Moderate to late preterm: 32 to 37 weeks GA.

*PRD* Pregnancy Related Diseases, *HDP* Hypertensive Disorders of Pregnancy, *GA* Gestational Age, *FGR* Fetal Growth Restriction, *ALAT* enzyme Alanine-Aminotransferase, *ASAT* enzyme Aspartate-Aminotransferase, *HELLP* Haemolysis, Elevated Liver enzymes, Low Platelets, *LDH* Lacto-Dehydrogenase, *DIC* Disseminated Intravascular Coagulation

### Study design

Potential participants will be informed about the study by their obstetric healthcare professional. They provide her with a brief explanation and the written study information. The healthcare professional also asks permission for the researchers to call the woman to further explain the study procedures, verify the in- and exclusion criteria (Table 1), answer any uncertainties and emphasise that participation is completely voluntary.

**Fig. 1:**
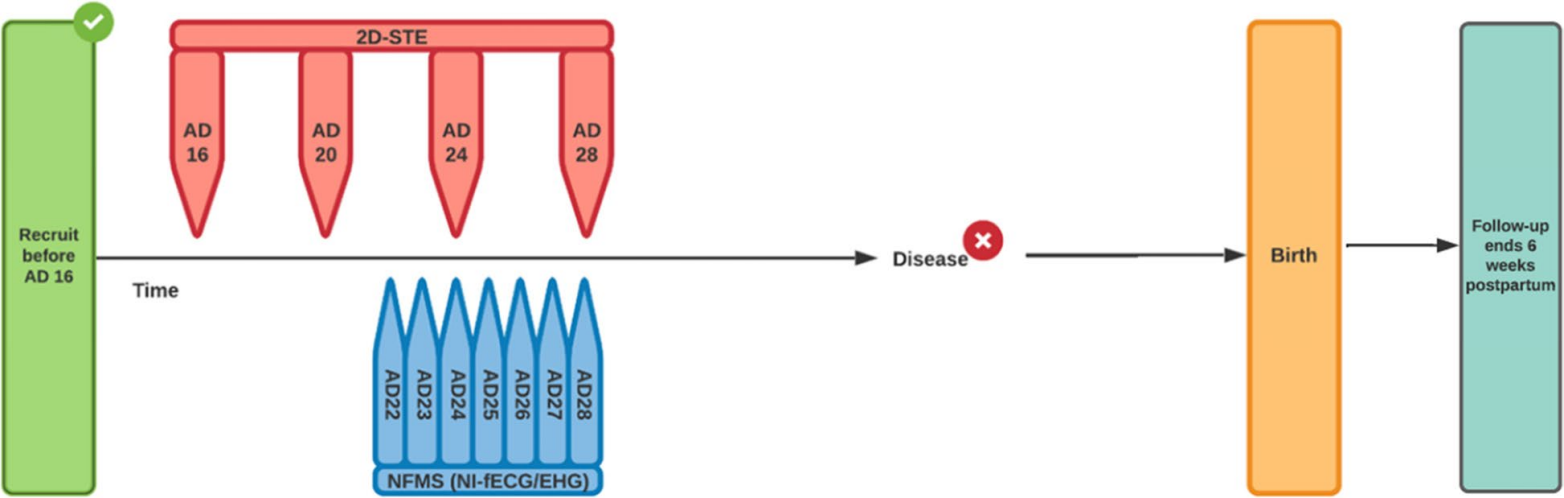
Inclusion will take place before 16 weeks GA. 2D-STE will be performed 4-weekly at 16, 20, 24 and 28 weeks GA. Weekly NI-fECG and EHG recordings will be made from 22 until 28 weeks GA. Follow up ends six weeks after delivery

Once a woman is included, 2D-STE measurements will be performed 4-weekly at 16, 20, 24 and 28 weeks GA. Weekly NI-fECG and EHG recordings will be made from 22 until 28 weeks GA. Follow up ends six weeks after delivery (Fig. 1).

The interval for 2D-STE, NI-fECG and EHG measurements are chosen to generate clear and specific reference values. As opposed to the weekly NI-fECG and EHG measurements, a 4-weekly evaluation is chosen for 2D-STE because of the small size of the fetal heart; measurement errors would probably have an undesirable large impact if the interval is narrowed. The chosen interval is in line with previous research in the field [24, 25].

### 2D-STE parameters

To analyse fetal cardiac remodelling, a competent sonographer acquires a four-chamber view of the fetal heart. A sonographer is considered competent after completing a training course where standard operating procedures are taught by an experienced obstetrician. A Philips EPIQ 7 ultrasound system will be utilized (Royal Philips N.V., Amsterdam, The Netherlands), including an appropriate ultrasound probe generating high frame rates (≥80Hz) to acquire sufficient quality of Digital Imaging and Communications in Medicine (DICOM) images for analysis [16, 17]. Using the right settings, including sector width, depth and zoom box is essential to achieve high frame rates. If the four-chamber view is properly obtained, a DICOM clip of at least three complete heart cycles will be saved. Data are acquired during fetal rest, with the pregnant woman holding her breath to avoid as many artefacts as possible.

Analysis for 2D-STE will be performed offline by independent and experienced researchers blinded to PRD. The DICOM clips will be analysed using the offline 2D Cardiac Performance 1.2 software (TomTec Imaging Systems GmbH, Munich, Germany).

The following 2D-STE parameters will be investigated in this study to assess fetal cardiac remodelling: global longitudinal strain, global longitudinal strain rate, velocity, dyssynchrony, sphericity index and shortening fraction [26]. However, global longitudinal strain and global longitudinal strain rate are considered to be the most realistic in the small fetal heart [16].

It has always been assumed that 2D-STE is an angle independent modality [16]. However, recent research indicates that insonation angle significantly influence fetal global longitudinal strain [27]. Because of some major shortcomings of this study, first additional evidence should be acquired to clarify the actual effect of insonation angles on fetal global longitudinal strain. In anticipation thereof, for every STE measurement three different DICOM clips will be generated corresponding to the various angles of insonation. Insonation angles will be defined as the angle between the ultrasound beam and fetal intraventricular septum. They will be divided into three groups: apex up or down (0° ± 22° or 180° ± 22°), apex oblique (45° ± 22°, 135° ± 22°, 225° ± 22° or 315° ± 22°) and apex perpendicular (90° ± 22° or 270° ± 22°) [27].

### NI-fECG and EHG parameters

Weekly NI-fECG and EHG measurements will be performed using a NFMS (Nemo Healthcare®, Veldhoven, The Netherlands). The system is CE-licensed and already in use as standard care in several hospitals throughout Europe.

**Fig. 2:**
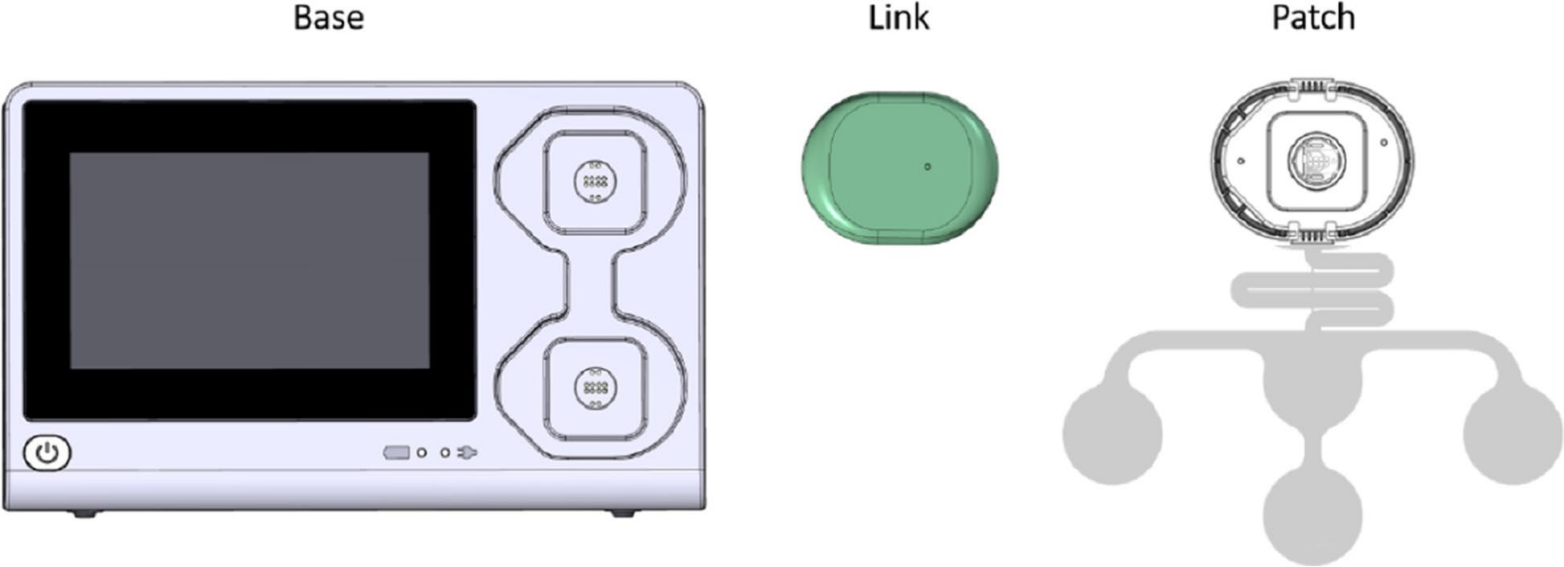
The NFMS consists of three components: base, link and patch (source: user manual Nemo Fetal Monitoring System®)

**Fig. 3:**
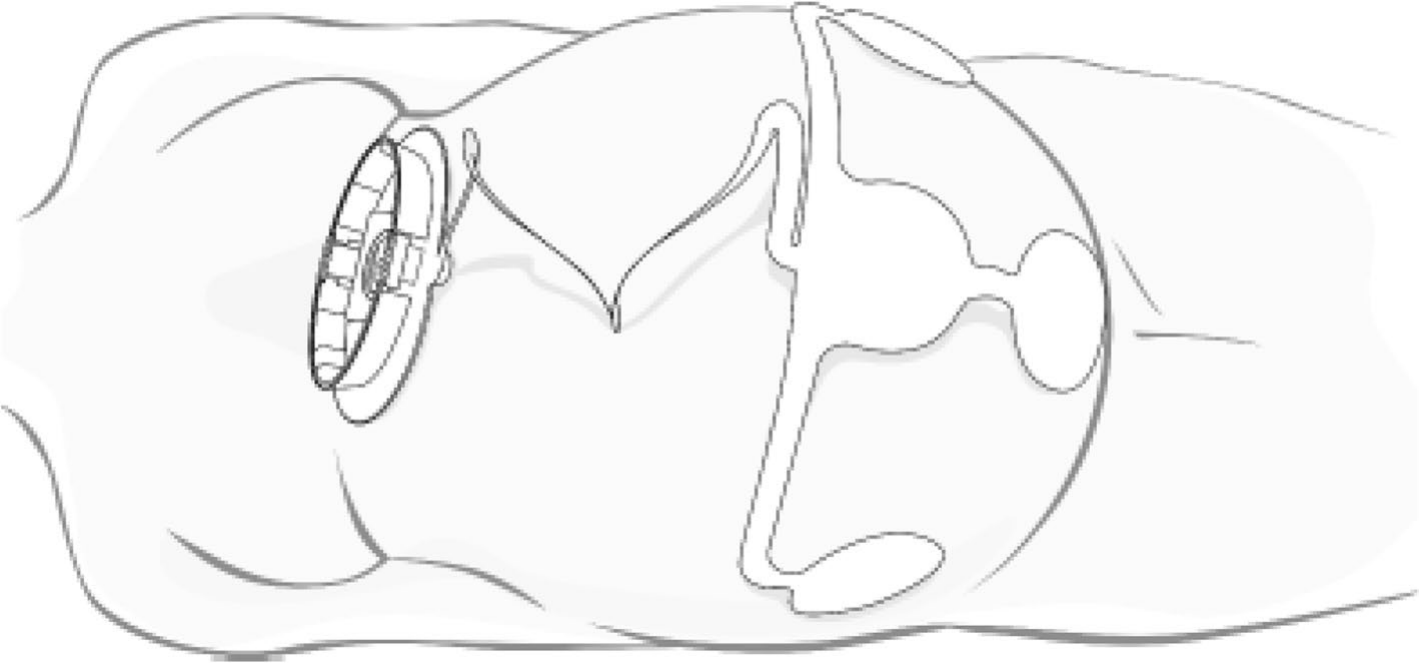
Placement of the abdominal patch. In all places where an electrode is placed, the skin must first slightly be abraded to achieve low skin impedance (source: user manual Nemo Fetal Monitoring System®)

The NFMS consists of a base, link and abdominal patch, including six electrodes (Fig. 2). Participants will be lying down in a semi-recumbent position during the NI-fECG and EHG recording. The electrode patch will be applied by trained staff according to the instructions of the manufacturer; the patch will be placed within the boundaries of the uterus (Fig. 3). After washing the abdominal skin with water and soap, the skin must be slightly abraded with medical abrasive paper. This step is crucial to achieve low skin impedance, ensuring good electrical conduction of the fetal heart signal [28]. The optimal cut-off value for skin impedance depends on the device that is used for NI-fECG measurements. Therefore, signal conduction quality will be indicated by the device with “good”, “suboptimal” or “bad”. When impedances are suboptimal or bad, skin abrasion can be repeated once. Every recording will last for 40 minutes. Researchers and subjects are blinded to the NI-fECG and EHG recordings and will be analysed retrospectively after childbirth.

Indexes of NI-fECG that will be analysed are fHRV in both time and frequency domain and cardiac time intervals (CTI). Time-domain indexes quantify the amount of variability between successive heartbeats [29, 30]. Frequency domain indexes estimate the distribution of power into the different frequency bands. The low-frequency (LF) power component of fHRV (0.04–0.15 Hz) is often used as a reflection of sympathetic activity. High-frequency (HF) power (0.4-1.5 Hz) represents parasympathetic activity. The ratio between LF and HF is regularly used to describe sympathetic-parasympathetic balance [30, 31].

The fetal heart grows proportionally with the complete fetal body and the time an electrical signal needs to travel before it can depolarize the muscle fibres is related to the size of the fetal heart [32]. With this in mind, CTI can potentially reflect FGR or cardiac remodelling [33]. CTI, such as PR-, QT-, PQ-, ST- or QRS intervals, will therefore also be obtained from the NI-fECG.

To conduct a dependable NI-fECG complex, it is essential to take into account the moving fetus and according changes in the fetal heart axis. Therefore, fetal orientation will be defined using an abdominal ultrasound at the beginning of every NI-fECG recording. The researchers are capable to track fetal movements from this point onwards based on the fetal heart electrical signal. To ensure a correct fetal position measurement, the fetal position measurement will be repeated once more. Fetal orientation will be documented in a digital fetal position annotator.

Description of CTG waveforms will be similar to the Fédération Internationale de Gynécologie et d'Obstétrique (FIGO) classification and includes baseline heart frequency, variability and reactivity [34].

For EHG, indexes that will be analysed are contraction frequency, duration, amplitude, conduction velocity and entropy.

All 2D-STE, NI fECG, and EHG measurements will be performed by trained researchers at the outpatient clinic of the tertiary care hospital. All other required demographic and medical information will be obtained from the prospective collected electronic patient files at the tertiary care hospital or the primary midwifery practice. This includes maternal information: age, ethnicity, body mass index, obstetric history, general medical history, medication use, intoxications, information about the course of pregnancy, complications during pregnancy, delivery or postpartum, ultrasound results such as fetal growth, structural abnormalities or Doppler measurements. Neonatal information: gender, birthweight, admission to neonatal ward with reason, Apgar scores, pH in umbilical artery and vein, mortality, general information on neonatal health in the first 6 weeks postpartum. Research Manager (ISO 27001 certified) is used to store patient data.

### Sample size

A total of 200 healthy participants would be sufficient to generate reliable reference values; precision would only slightly increase by including additional participants [35]. This follows from the formula to calculate the standard error:

$$SE= SD\ast \sqrt{3/n}$$

However, this number of participants (*n*=200) will not be adequate to answer the secondary objective. For that reason, sample size numbers are based on the secondary outcomes.

**Fig. 4:**
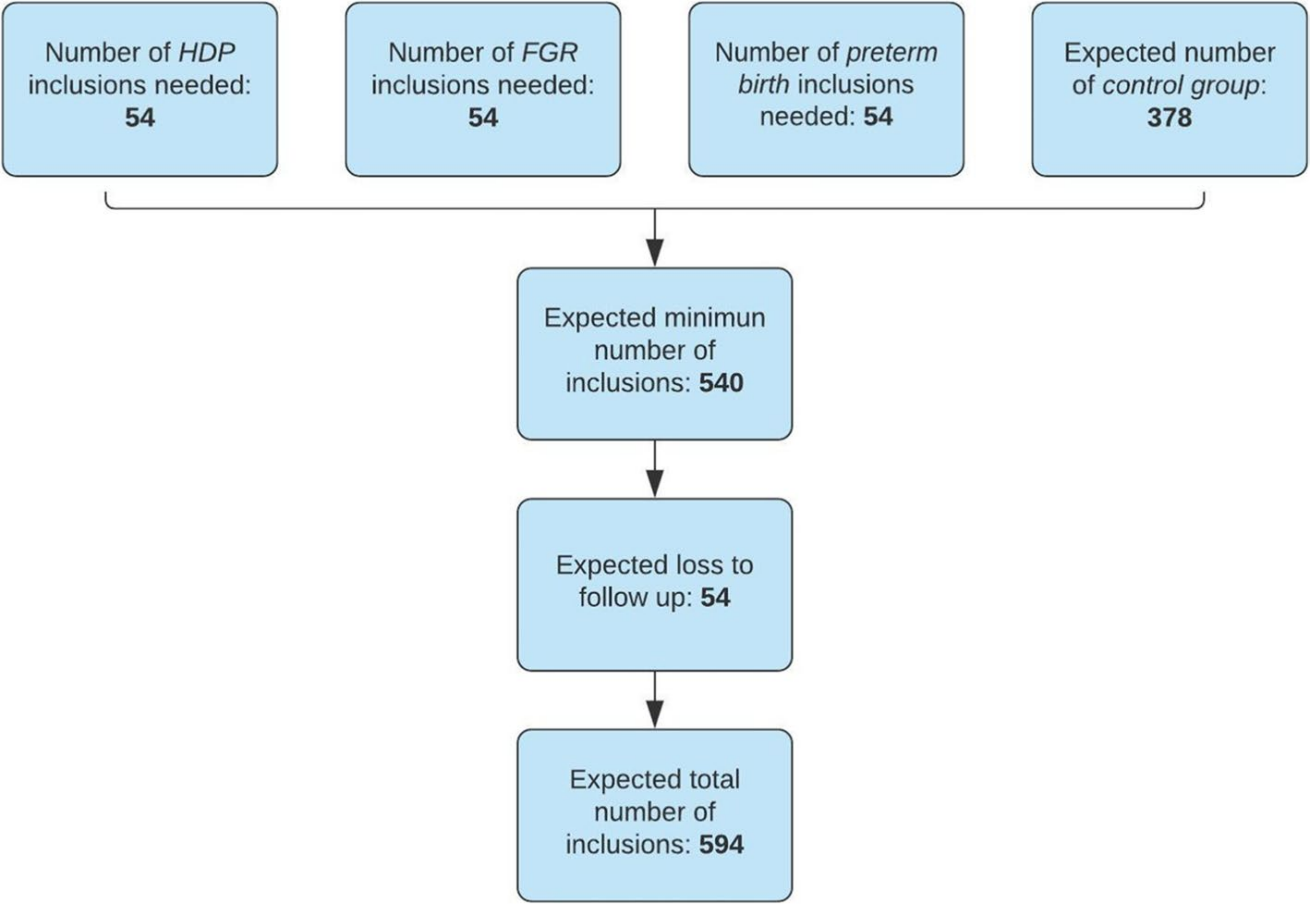
Flow chart of sample size calculation

Incidence for HDP is up to 15% [36], up to 10% for FGR [37] and up to 18% for preterm birth [2]. Based on very limited previous research on the effect of HDP on 2D-STE, an effect size of 0.4 could be calculated [18]. We used G*Power 3.1 software to calculate the sample size using a two-tailed test with a power of 0.8, an effect size of 0.4, alpha 0.05 and an allocation ratio of 10. This results in 54 participants for each PRD group, respectively. To achieve this, we will include 540 participants in the cohort, given the PRD incidence of at least 10%. Anticipating on 10% loss to follow-up, 594 participants are expected to be included in the cohort. Inclusions will continue until 54 participants are included in each PRD group (Fig. 4).

### Statistical analysis

The population on which the reference values will be based consists of all women with a healthy pregnancy. Parametric statistics will be used to describe normally distributed data; otherwise non-parametric statistics will be used, including 95%-confidence intervals. The linear mixed model will be used. This method accounts for measurements in which all patients are measured repeatedly at the same time intervals. If needed, multiple imputation will be applied for missing data to retain power.

Secondarily, we will investigate the diagnostic potential of 2D-STE, NI-fECG and EHG. To examine this, a logistic regression model will be applied to model the binomial outcome data to covariates. It is our intention to estimate the impact of each variable on the odds ratio of the observed event of interest. This will be achieved by performing a multivariate regression analysis including all variables that are associated with the outcome based on either existing literature or data of this study. To reduce the risk of overfitting, a backward elimination procedure will be performed and information criteria will be used. Possible multicollinearity will be investigated using the variance inflation factor. If multicollinearity exist, it will then be considered to centre the data or remove a variable from the logistic regression model. A receiving operating characteristic (ROC) curve will be generated using the probabilities as calculated with the multivariate regression model to calculate the area under the curve and define the optimal cut-off value for 2D-STE, NI-fECG and EHG measurements correlated with PRD. Sensitivity, specificity, positive- and negative predictive value with 95% confidence intervals will be calculated for this cut-off.

It is important to recognize that HDP and FGR have overlapping pathogenesis. For that reason, the FGR cases will be excluded for analysis of the HDP group and vice versa to secure an unadulterated comparison [38]. A *p*-value of <0.05 is considered statistically significant for all statistics.

### Interim analysis

An interim analysis will be performed after 20 participants completed all measurements for this study. The main purpose of this analysis is to assess the quality of the NI-fECG and EHG measurements, since abdominal patches will be reused for repeated measurements within the same participant. If the quality turns out to be insufficient for analysis, we will no longer reuse the patches but apply a new patch for each NI-fECG and EHG measurement. Participants with insufficient data will be excluded and replaced by another woman.

## Discussion

All technical resources investigated in this study might be very helpful to detect PRD at a very early stage of pregnancy and can potentially prevent serious complications by early detection of PRD. However, it is a very novel area and more extensively research is required before it can be implemented in daily practice. Due to the comprehensiveness of this study, the final database can also be used as a reference database for future research.

The definition of FGR as used in this study may cause controversy since the individualized growth potential is not taken into consideration. Consequently, large fetuses that did not reach their growth potential can be missed as FGR whereas constitutionally small fetuses will possibly be over diagnosed. Dynamic definitions, which include pulsatility index of the umbilical and uterine artery, are compiled trying to incorporate this individual growth potential [39]. However, literature disagrees on the advantage of a dynamic definition on clinical outcome [40]. Moreover, research showed signs of prenatal cardiac dysfunction in constitutionally small fetus, indicating that at least a proportion of them is not just “constitutionally” small [41, 42].

The Hadlock ultrasound measurements are the best available antepartum method to estimate fetal growth. However, ultrasound measurements are sensitive to significant measurement errors in the diagnosis of FGR [43]. For that reason, the antepartum definition according to the Dutch guidelines [44], will not be adhered to for this study. Instead, birthweight corrected for GA will be used [22].

The 2D-STE is highly sensitive and capable of detecting preclinical myocardial deformations in adults [45]. Previous research identified echocardiographic cardiac changes without deviations in conventional ultrasound measurements in the fetus [18]. This implicates that cardiac remodelling is first in the pathological pathway and could be a superior method for early detection of PRD in the future. In previous research, significantly deviating results for cardiac deformation in healthy pregnancies compared to pregnancies complicated by HPD^19^ or FGR [20] were found. Though, due to great heterogeneity more research is fundamental.

NFMS consists of NI-fECG and EHG measurements, which together generate a CTG [46]. Nowadays, antepartum CTG is generated by DU and TOCO to determine fetal wellbeing. However, CTG measured by DU and TOCO faces some major disadvantages; both are adversely influenced by maternal obesity and maternal and fetal movements. Signal loss of the fetal heart rate may occur often, inflicting frequent relocation of the DU transducer. Unlike DU and TOCO, NI-fECG and EHG are less influenced by maternal obesity [47]. Also, higher sensitivity and reliability of NI-ECG and EHG compared to DU and TOCO was found in literature [48, 49]. Therefore, NI-fECG and EHG are promising alternative non-invasive monitoring methods for antepartum assessment of fetal and maternal wellbeing.

Additionally, NI-fECG and EHG enables possibilities for detection of PRD as early as the second trimester. The NFMS measures NI-fECG as well as EHG allowing for analysing of every consecutive fetal ECG complex as well as electrical uterine activity.

As mentioned in the introduction, fHRV is an important indicator to assess fetal wellbeing [50]. The FIGO classification already assesses this parameter, though the widely used DU uses averaged heart rate frequency on which medical professionals can only interpret gradations of fHRV [34]. In contrast, the application of spectral analysis (used to distil LF and HF frequency power) on the consecutive fetal ECG complexes gives very specific information about the functioning of both branches of the autonomic nervous system. Thus, fHRV assessed by DU is less accurate and less detailed compared to the real-time variability assessed by NI-fECG. We hypothesise that fHRV decreases in women diagnosed with HDP and FGR.

Literature shows that fetal movements affect fHRV gradually during gestation [51, 52]. However, fetal behavioural states have only been observed from 33 weeks GA and cannot be distinguished in the second trimester [53]. Other types of fetal movements, like respiratory sinus arrhythmias and fetal gross motor activity, have only shown a relevant effect on accelerations from 30 weeks GA [54, 55]. In the second trimester, minimal differences in fetal heart rate characteristics between the active and inactive fetus have been observed [55]. For this reason, fetal movements are not taken into account for this study.

CTI is another parameter that cannot be derived from contemporary CTG using DU, but will be analysed in our study. We hypothesise that CTI decreases as a sign of placental dysfunction and the resulting cardiac deformations.

Previous research showed promising results of some EHG parameters to predict preterm deliveries [11, 56, 57]. Most of the studies were based on the Term-Preterm EHG Database, a public database of EHG recordings during regular check-ups containing both term and preterm deliveries [57]. However, many different measurement properties are used in literature. To the best of our knowledge, normal values of EHG parameters during uncomplicated pregnancy are lacking. We expect to demonstrate differences in EHG parameters in women who will deliver preterm and women who deliver term; entropy might be smaller in women delivering preterm, whereas contraction frequency, intensity and velocity might be higher. By identifying these differences early in gestation, we aim to diagnose and predict preterm birth better in the future.

### Study limitations

Between 28 and 32 weeks GA, the fetus is covered in a fatty layer, the vernix caseosa, which may cause disturbances in the fetal cardiac signal and which can therefore lead to insufficient data for analysis of NI-fECG [28]. However, most measurements will be performed before the vernix period. Yet, a poor signal will not be noticed by the researcher during the measurement, because the screen of the NFMS is shielded. The researcher is therefore unable to adjust the abdominal patch to improve signal quality.

Accurate determination of the fECG amplitudes may be subject to large inter-observer variability. This can complicate the defining of CTI. The premature GA and the corresponding small size of the fetal heart in combination with large signal noise can predominate the small repolarization signal of the fetal atria. Band-pass filters incorporated in the NFMS are supposed to filter most signal noise and make the fetal signal as clear as possible.

### Ethics and risk assessment

For correct application of the NFMS patch, only a negligible chance of mild skin irritation or a minor allergic reaction caused by abrading the abdominal skin or applying conduction gel can be expected. However, some other troublesome situations should be explained carefully. In current practice, decisions on the need for obstetric interventions are primarily based on ultrasounds measurements and CTG parameters similar to the parameters collected in this study with the NFMS. However, in the non-viable period, it is very unusual to execute this examination as there is yet no clinical action indicated in case of abnormal findings. Moreover, it is unknown if reference values for term fetuses can be applied to fetuses during the preterm period.

In the Netherlands, the non-viable period is defined as pregnancies before 24 weeks GA. For this study, NI-fECG and EHG measurements are conducted as early as 22 weeks GA. By blinding the NI-fECG and EHG measurements, the researchers assure that clinical decisions are based solely on standard care and possible ethical dilemmas are evaded.

Nevertheless, generating the four-chamber view of the fetal heart cannot be blinded. In the unlikely event of an unexpected finding, the woman and her obstetric healthcare professional will be informed immediately. Depending on the seriousness of the finding and its implication on the fetal heart, the woman will be excluded from the cohort and replaced by another woman.

## Data Availability

Data sharing is not applicable to this article as no datasets were generated or analysed during the current study.

## Abbreviations

ALAT: Enzyme aspartate-aminotransferase
ASAT: Enzyme alanine-aminotransferase
CRH: Corticotropin Releasing Hormone
CTG: Cardiotocography
CTI: Cardiac Time Intervals
DIC: Disseminated Intravascular Coagulation
DICOM: Digital Imaging and Communications in Medicine
DU: Doppler Ultrasound
EHG: Electrohysterography
FGR: Fetal Growth Restriction
fHRV: fetal Heart Rate Variability
FIGO: Fédération Internationale de Gynécologie et d'Obstétrique
GA: Gestational Age
HDP: Hypertensive Disorders of Pregnancy
HELLP: Haemolysis, Elevated Liver enzymes, Low Platelets
HF: High Frequency
LDH: Lacto-Dehydrogenase
LF: Low Frequency
NFMS: Nemo Fetal Monitoring System
NI-fECG: Non-Invasive fetal Electrocardiography
PRD: Pregnancy-Related Diseases
ROC: Receiving Operating Characteristic
SE: Standard Error
SD: Standard Deviation
TOCO: External Tocodynamometry
2D-STE: Two-Dimensional Speckle-Tracking Echocardiography
11β-HSD: 11 Beta Hydroxysteroid Dehydrogenase
